# Evaluation of the anesthetic effect of epinephrine-free articaine and mepivacaine through quantitative sensory testing

**DOI:** 10.1186/s13005-015-0061-1

**Published:** 2015-02-07

**Authors:** Sareh Said Yekta-Michael, Jamal M Stein, Ernst Marioth-Wirtz

**Affiliations:** Department of Conservative Dentistry, Periodontology and Preventive Dentistry, Aachen University, Aachen, North Rhine-Westphalia Germany; Interdisciplinary Center for Clinical Research, RWTH Aachen University, Aachen, North Rhine-Westphalia Germany

**Keywords:** Articaine, Mepivacaine, Local anesthesia, Maxillary infiltration, Trigeminal region, Epinephrine-free

## Abstract

**Introduction:**

Long lasting anesthesia of the soft tissue beyond the dental treatment affects patients in daily routine. Therefore a sophisticated local anesthesia is needed. The purpose of this study was an evaluation of the clinical use of epinephrine-free local anesthetic solutions in routine short-time dental treatments.

**Materials and methods:**

In a prospective, single-blind, non-randomized and controlled clinical trial, 31 patients (16 male, 15 female patients) undergoing short-time dental treatment under local anesthesia (plain solutions of articaine 4% and mepivacaine 3%) in area of maxillary canine were tested with quantitative sensory testing QST. Paired-Wilcoxon-testing (signed-rank-test) and Mc Nemar tests have been used for statistical results.

**Results:**

Significant differences in all tested parameters to the time of measurements were found. Mepivacaine showed a significantly stronger impact for the whole period of measurement (128 min) on thermal and mechanical test parameters and to the associated nerve fibers.

**Conclusion:**

Plain articaine shows a faster onset of action associated with a shorter time of activity in comparison to plain mepivacaine. In addition to this articaine shows a significant low-graded effect on the tested nerve-fibers and therefore a least affected anesthesia to the patient. The clinical use of an epinephrine-free anesthetic solution can be stated as possible option in short dental routine treatments to the frequently used vasoconstrictor containing local anesthetics. Patients may benefit from shorter numbness.

## Introduction

In dental treatment, the management of pain is an important factor for patient comfort and a critical element of patient care [1,2]. A huge amount of local anesthetic agents are used nowadays, but only a few of these available agents have been exposed to be more beneficial in dental routine. These few compounds, which mostly contain a vasoconstrictor e.g. epinephrine, are widely used [3]. The clinical benefit of local anesthetics combined with vasoconstrictors is obvious: local ischemia is provided by the supplement in the region of attention, which extends the duration activity and reduces the systemic toxicity of the anesthetic agent [4,5].

However, there are also clinical situations, which do not allow the use of agents containing vasoconstrictors, particularly epinephrine. Clinical findings and contraindication in anamnesis are e.g. hypertension, hyperthyreosis, diabetes mellitus and all advice of vasoconstrictions [6-11]. In order to avoid systemic adverse reactions, local anesthetic solutions should always contain the minimum concentration of epinephrine [4]. It is very important for the clinician to be acquainted with the characteristics of the agent. Especially pharmacological action, toxicity and maximal dose are important parameters to know.

In addition attention has to be payed to the onset, the depth of anesthesia and the duration of activity related to the planned dental procedure. Unfortunately the compounds, which are commonly used and contain a vasoconstrictor, have a disadvantage, especially concerning routine nonsurgical short-time dental treatments. There is a discrepancy between the time for dental procedures and the time of agent’s activity, especially on patient’s soft tissue [4]. Several studies have proved that soft tissue anesthesia can last for 3 – 4 hours beyond the treatment when an agent with vasoconstrictor was used [12,13]. In particular the long-lasting anesthesia of soft tissue, causing unpleasant numbness beyond the dental treatment, affects the patient in daily routine. Eminently because of this negative effect on patient’s comfort, local anesthesia has to be justified for routine short-time dental treatments.

A situation-related compound, which approaches the possibility of a safe and adequate dental care and the need of the patient, meaning a least affecting anesthesia, has to be chosen [4,14]. Studies have confirmed that plain solutions of local anesthesia provide an alternative option to the commonly used compounds [14]. Although a variety of plain local anesthetic solutions are available, they are rarely used in daily routine. The plain solutions Mepivacaine shows the typical structure of an amino-amide local anesthetic and was object of different studies [15]. In contrast to other local anesthetics of the amino-amid type, plain mepivacaine shows vasoconstrictor-properties at its disposal [15,16].

Articaine is also a local anesthetic of an amide type. But since it possesses vasodilatation-properties [16], it is mostly used in association with a vasoconstrictor increasing the local anesthetic efficacy. The characteristic property of articaine is its chemical structure: a thiophene-ring in addition to an ester linkage. This chemical structure represents an exception in the line of local anesthetics in clinical use [1]. Several studies have exposed that articaine is the local anesthetic agent with the best properties of diffusion within soft and hard tissue [4].

In order to test the effect of soft tissue-anesthesia and in particular the effect of the compound being used on sensory modalities of large and small fibers (Aβ-, C- and Aδ- fibers), a reliable noninvasive psychophysical test-method can be performed; the quantitative sensory testing (QST). This QST procedure has become an integrable diagnostic tool in clinical routine with a standardized battery of thermal and mechanical parameters [17,18]. It has already been used successfully in the trigeminal region of face [17-21]. In the present study QST was adapted to the hairy skin of the infraorbital region (V2) during small dental interventions in maxillary premolares.

The aim of the current study is to elevate the clinical use of vasoconstrictor-free local anesthetics in routine short-time dental treatments and especially to discover the effect of the local anesthetic agents (articaine/mepivacaine) on sensory modalities of large and small fibers (Aβ-, C- and Aδ- fibers) and the differences of the resulting soft-tissue anesthesia. The purpose is to discover a situation-justifying agent, which can be used by the clinician for routine short-time dental treatments and also improves the situation of the patient due to the limited duration of soft-tissue anesthesia.

## Materials and methods

### Patients

Thirty-one patients (15 female, male 16) covering an age between 20 and 40 years (27.4 ± 4.0 years, mean ± SD) in need of small nonsurgical dental treatment in region of maxillary premolares were included in this prospective, controlled non-randomized, single blind study. Only patients, who fulfilled the inclusion criteria and none of the exclusion criteria, were tested.

The inclusion criteria involved the necessity of a dental filling therapy in maxillary premolar region, filling-free maxillary canines, the wish of the patient and the medical indication for a dental local anesthesia in the region of the dental treatment and furthermore the age of the patients (20 – 40 years). The exclusion criteria were all kinds of cardiovascular-, metabolic-, CNS-, immune system- disease; furthermore coagulopathy, circulatory disorder, recent operations, contagious diseases, sulfite sensitivity or allergy to any part of the solution, psychiatric illness, drug consumption and pregnancy. All patients have been without any medication for 48 hours.

All participants gave their informed consent prior to the inclusion in this study according to the 1964 declaration of Helsinki. The study took place at the department of conservative dentistry, periodontology and preventive dentistry of RWTH University Aachen. The protocol passed the local ethics committee (number of the acceptance by the ethic committee EK 076/11).

Thermal and mechanical detection and pain thresholds were determined by the quantitative sensory testing protocol (QST) [17,18,22]. In several studies it was recommended to reduce the originally QST protocol of 13 parameters to less parameters without affecting the informative value of the measurement in order to ensure an integration into clinical practice. With thermal and mechanical stimuli of QST it is possible to show distinct neuroanatomic pathways with Aβ, Aδ, and C fiber populations [21,23,24].

**Figure 1:**
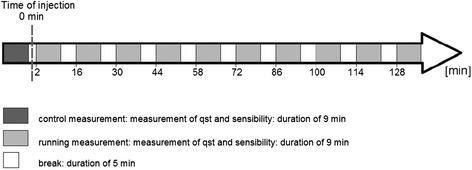
**Stimulation protocol.**

Because of the limited time of clinical routine it was necessary to design a test-protocol obtaining the sensory profiles within dental treatment. Therefore in the present study 7 of the 13 possible test parameters of QST were included into the test protocol; containing CDT, cold detection threshold; WDT, warm detection threshold; CPT, cold pain threshold; HPT, heat pain threshold; MDT, mechanical detection threshold; MPT, mechanical pain threshold and VDT, vibration detection threshold. In addition to that the pulp sensory of the canine was tested in a circle of 5 minutes for the whole test period by using a cold test, which seeks a response to the thermal stimulus (Figure 1).

### Clinical examination- treatment protocol

At the beginning of every test period a control test was implemented in order to demonstrate the test method to the patient and to collect normative data. For realizing a painless small dental treatment (fissure-filling-therapy in premolares on both sides of maxilla) a maxillary local infiltration anesthesia in canine region was performed. For that, plain solutions of articaine 4% (Ultracain D®, Sanofi-Aventis, Paris, France) and mepivacaine 3% (Meaverin®, Actavis, New Jersey, USA) were applied. The local anesthetics were given to all patients, alternately on the right and left side of maxilla; in conclusion every agent was nearly administered equal times on every side. Thereby an amount of 1.00 ml solution was given in 30 sec per dental syringe (Uniject k®, Fa. Sanofi-Aventis, Paris, France) always performed by the same dentist. Immediately after the injection the first test of the test-interval was performed; each test, containing QST and the test of pulp sensibility, took 9 min. During the five-minute breaks between every single test-interval, the dental treatment was performed. Patients were lying on a dental chair and kept their eyes closed throughout the QST procedure. Half of the group was tested on the right side first, the other half on the left side. All tests were performed by the same trained examiner.

### Quantitative sensory testing

The test protocol containing thermal and mechanical detection, as well as pain thresholds, was always applied in the same order. Thereby the patients were tested thermal before mechanical testing occured.

All thermal stimuli were assigned by a special computer-controlled peltier type thermode with a stimulation area of 16 × 16 mm^2^ (TSA-II, Medoc Ltd., Israel). Starting at a temperature-baseline of 32°C, the temperature of the thermode decreased or increased by 1°C/sec to determine thermal detection and pain thresholds (CDT, WDT, CPT, and HPT). The patients were asked to press a computer mouse button as soon as they observed the corresponding cold, warm, cold pain, or heat pain sensation. After receiving measurement results, the temperature of the thermode returned to baseline-temperature for each thermal threshold. In order to protect the patient’s skin, the range of stimulation temperatures was controlled between 0 – 50°C. The test-procedure for cold and warm detection thresholds (CDT, WDT) was performed firstly. Afterwards cold pain- and heat pain thresholds (CPT, HPT) were determined.

The mechanical test procedure contained the thresholds for MDT, MPT and VDT.

MDT was rated with modified von Frey filaments featuring forces of 0.08, 0.2, 0.4, 0.7, 1.6, 4, 6, 10, 14, 20, 40, 60, 80, 100, 150, 260, 600, 1.000, 1.800, 3.000 mN (Touch-Test Sensory Evaluators; North Coast Medical, Morgan Hill, CA, USA).

For performing the measurement of MPT, custom-made weighted pinprick stimulators with forces of 8, 16, 32, 64, 128, and 256 mN and a contact area of ca. 0.2 mm diameter were applied to hairy skin of the infraorbital region.

MDT and MPT were set by the method of limits starting with a clearly noticeable filament of 16 mN and a non-painful pinprick stimulator of 8 mN.

Both thresholds were defined as the geometric mean of 5 series of descending and ascending stimulus intensities [25,26].

The vibration stimuli were applied by a 64-Hz Rydel-Seifer tuning fork (OF033N; Aesculap, Tuttlingen, Germany) that was placed over the maxilla (infraorbital nerve area). Threshold measurement was performed 3 times starting with maximum vibration amplitude. As soon as the subject indicated disappearance of vibratory sensation, the threshold was read on a scale ranging from 0/8 to 8/8 (steps of 1/8). VDT was defined as the arithmetic mean of 3 runs [25,26].

### Statistical analysis

All statistics were done in an explorative manner; no adjustment of significance level for multiple testing was performed. The function of statistic was to investigate differences between the anesthesia-tread of both agents at testing time; furthermore to discover the differences between the measurement of control tests and continuing measurement tests. For all QST parameters differences between the agents were performed using the Wilcoxon-signed rank test. Testing the pulp sensibility the Mc Nemar Test was performed. Significance level was accepted at p < 0.05. Statistical analysis was done with SigmaStat 11.0 (SPSS Inc., USA) and SAS (SAS Institute Inc., USA).

## Results

Altogether, thirty-one patients, sixteen male and fifteen female, with a mean age of 27.4 ± 4,0 years (mean ± SD), a mean size of 173.6 ± 6.3 cm (mean ± SD) and a mean weight of 69,3 ± 10,6 kg (mean ± SD) could be included.

### Thermal testing

**Figure 2:**
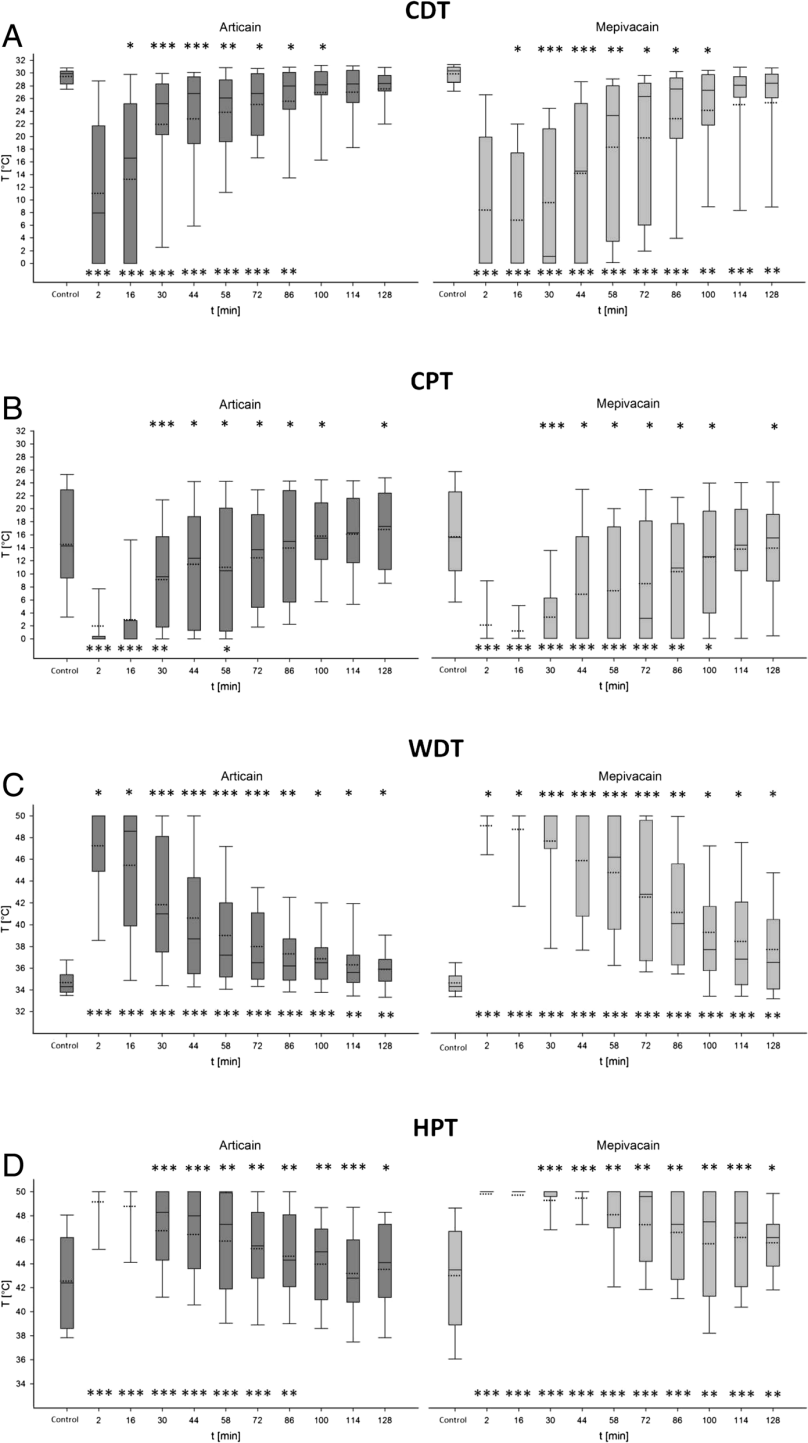
**Presentation of the results of the thermal QST parameters (A: CDT Articain, CDT Mepivacain; B: CPT Articain, CPT Mepivacain; C: WDT Articain, WDT Mepivacain D: HPT Articain, HPT Mepivacain) and the results of the statistical analysis for the individual measurement points (inclusive standard deviation).** Significant differences are indicated by asterisks (paired Wilcoxon test: p *** ≤ 0.001; ** p ≤ 0.01, p ≤ 0.05 *, ns = not significant). The upper asterisks indicate significant differences between the measured values of articaine and mepivacaine to the respective measurement time. The lower asterisks indicate significant differences between the control measurement value and the running values of each anesthetic. Median value (solid line) and mean value (dotted line) are shown within the boxplots.

The effect of both anesthetic agents showed significant differences at each time of measurement for CDT as well as for WDT (Table 1, Figure 2). Equivalent amounts of the two local anesthetics exerted a different effect on the intense temperature-detection thresholds. Mepivacaine showed a stronger influence (p < 0.05) on the temperature sensitivity across all points of measuring, as well as higher detection thresholds (p < 0.05) in CDT and WDT than articaine. For both, CDT (p < 0.001) and WDT (p < 0.01), the values of control measurement were not achieved until the end of measurement progress. Until the last point of measurement (128th minute) a significant difference between the control measurement and the course measurements existed. Moreover, significant differences in anesthesia profiles occured in CPT as well as in HPT. By comparing the analysis of the control value and the course measurements an end of significant differences for cold pain threshold (CPT) was reached within the measurement period in both preparations. Articaine reached the value of control measurement earlier (t = 72 min, p > 0.05) than mepivacaine (t = 100 min, p > 0.05). Only at heat pain threshold (HPT) no significant differences were registered for articaine from the point of mensuration (t = 100 min, p > 0.05) to the received value of the control measurement. Even CPT and HPT showed that mepivacaine had a greater effect on thermal pain thresholds (p < 0.05) and maintained this throughout the measuring time.

**Table 1:** **Measurement results of the QST thermal testing (CDT, CPT, WDT, HPT)**

	**CDT**					**CPT**					**WDT**					**HPT**				
	**Articaine 4%**		**Mepivacaine 3%**			**Articaine 4%**		**Mepivacaine 3%**			**Articaine 4%**		**Mepivacaine 3%**			**Articaine 4%**		**Mepivacaine 3%**		
**Time of measurement [min]**	**median [°C]**	**p-value control vs. course**	**median [°C]**	**p-value control vs. course**	**p-value articaine vs. mepivacaine**	**median [°C]**	**p-value control vs. course**	**median [°C]**	**p-value control vs. course**	**p-value articaine vs. mepivacaine**	**Median [°C]**	**p-value control vs. course**	**Median [°C]**	**p-value control vs. course**	**p-value articaine vs. mepivacaine**	**Median [°C]**	**p-value control vs. course**	**Median [°C]**	**p-value control vs. course**	**p-value articaine vs. mepivacaine**
**Control- measurement**	29,90	-	30,30	-	-	14,30	-	15,50	-	-	34,30	-	34,30	-	-	42,40	-	43,50	-	-
**2**	7,90	<0,001	0,00	<0,001	0,346	0,00	<0,001	0,00	<0,001	0,85	50,00	<0,001	50,00	<0,001	0,042	50,00	<0,001	50,00	<0,001	0,156
**16**	16,60	<0,001	0,00	<0,001	0,036	0,00	<0,001	0,00	<0,001	0,129	48,60	<0,001	50,00	<0,001	0,021	50,00	<0,001	50,00	<0,001	0,074
**30**	25,20	<0,001	1,10	<0,001	<0,001	9,60	0,005	0,00	<0,001	<0,001	41,00	<0,001	50,00	<0,001	<0,001	48,30	<0,001	50,00	<0,001	<0,001
**44**	26,80	<0,001	14,50	<0,001	<0,001	12,40	0,070	0,00	<0,001	0,022	38,70	<0,001	50,00	<0,001	<0,001	48,00	<0,001	50,00	<0,001	<0,001
**58**	26,10	<0,001	23,30	<0,001	0,004	10,50	0,029	0,00	<0,001	0,047	37,20	<0,001	46,20	<0,001	<0,001	47,30	<0,001	50,00	<0,001	0,007
**72**	26,80	<0,001	26,30	<0,001	0,018	13,70	0,139	3,10	<0,001	0,02	36,50	<0,001	42,80	<0,001	<0,001	45,50	<0,001	49,60	<0,001	0,006
**86**	28,00	0,003	27,50	<0,001	0,034	15,00	0,631	10,80	0,006	0,013	36,20	<0,001	40,10	<0,001	0,002	44,30	0,007	47,30	<0,001	0,008
**100**	28,20	0,012	27,30	<0,001	0,011	15,50	0,822	12,50	0,094	0,011	36,50	<0,001	37,70	<0,001	0,016	45,00	0,052	47,50	0,002	0,008
**114**	28,30	0,004	28,10	<0,001	0,245	16,30	0,524	14,30	0,411	0,053	35,60	0,003	36,80	<0,001	0,041	42,80	0,376	47,40	<0,001	<0,001
**128**	28,40	<0,001	28,40	<0,001	0,36	17,30	0,123	15,40	0,493	0,037	35,90	0,006	36,50	0,002	0,024	44,10	0,102	46,20	0,003	0,013

### Mechanical testing

**Figure 3:**
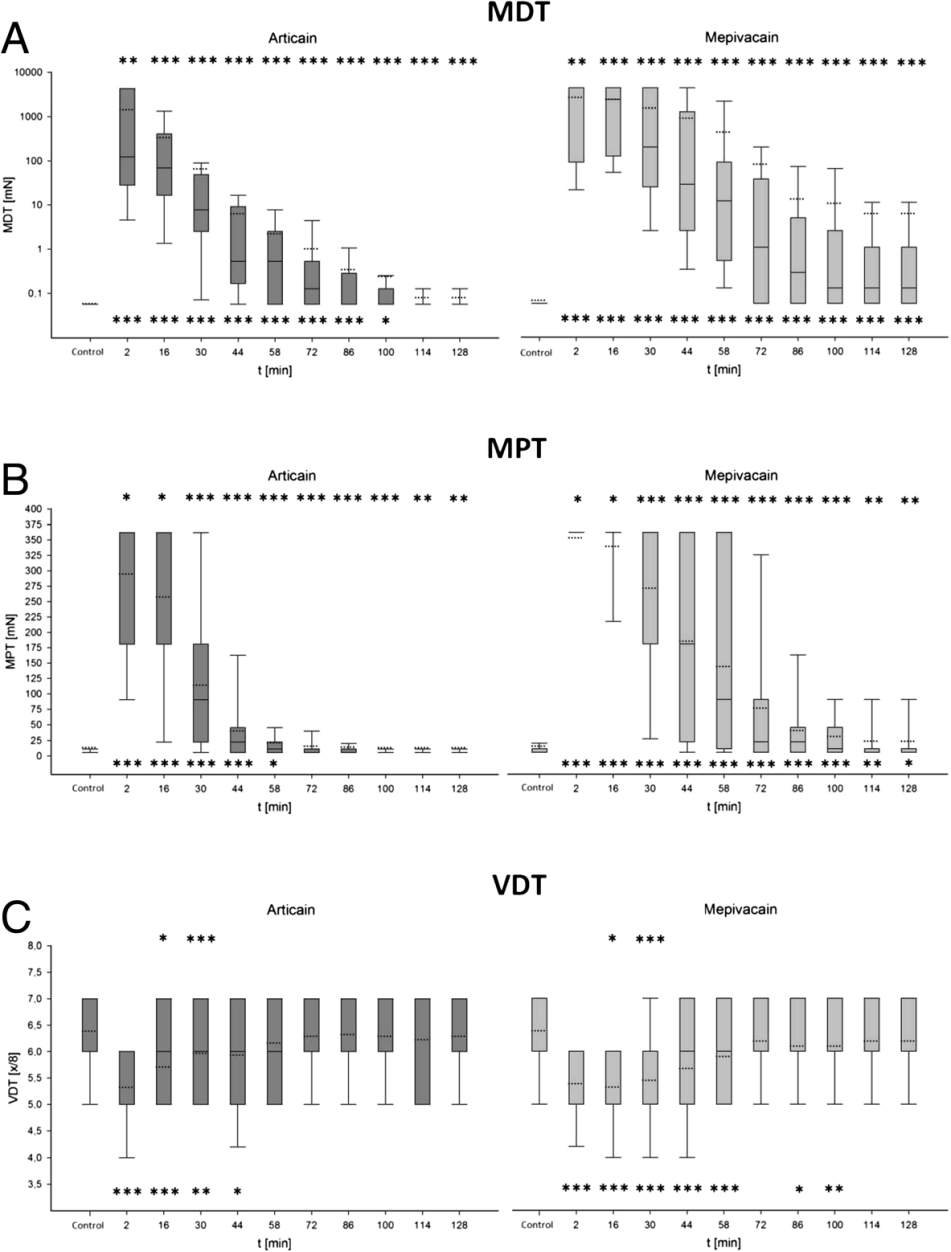
**Presentation of the results of the mechanical QST parameters (A: MDT Articain, MDT Mepivacain; B: MPT Articain, MPT Mepivacain; C: VDT Articain, VDT Mepivacain) and the results of the statistical analysis for the individual measurement points (inclusive standard deviation).** Control measurement and anesthesia course of the two local anesthetics in direct comparison. Significant differences are indicated by asterisks (paired Wilcoxon test: p *** ≤ 0.001; ** p ≤ 0.01, p ≤ 0.05 *, ns = not significant). The upper asterisks indicate significant differences between the measured values of articaine and mepivacaine to the respective measurement time. The lower asterisks indicate significant differences between the control measurement value and the running values of each anesthetic. Median value (solid line) and mean value (dotted line) are shown within the boxplots.

For MDT as well as MPT, significant differences between the measured sensitivity thresholds were recorded at each time of testing (Table 2, Figure 3). At the tactile detection threshold MDT the measurement values of the control measurement were not reached in mepivacain over the entire measurement time course (p < 0.05). At the local anesthetic articaine a comparison with the course of measurements shows that the biggest interference of MDT was directly after the injection. In following development sensitivity of the skin returned steadily. Up to 100th Minute, a significant discrepancy between the control measurement and course measurements were demonstrated (p < 0.05). From the 114th minute no significant difference with the control measurement consisted (p > 0.05).

**Table 2:** **Measurement results of the QST mechanical testing (MDT, MPT, VDT)**

	**MDT**					**MPT**					**VDT**				
	**Articaine 4%**		**Mepivacaine 3%**			**Articaine 4%**		**Mepivacaine 3%**			**Articaine 4%**		**Mepivacaine 3%**		
**Time of measurement [min]**	**Median [mN]**	**p-value control vs. course**	**Median [° mN]**	**p-value control vs. course**	**p-value articaine vs. mepivacaine**	**Median [mN]**	**p-value control vs. course**	**Median [mN]**	**p-value control vs. course**	**p-value articaine vs. mepivacaine**	**Median [x/8]**	**p-value control vs. course**	**Median [x/8]**	**p-value control vs. course**	**p-value articaine vs. mepivacaine**
**Control- measurement**	0,06	-	0,06	-	-	5,66	-	5,66	-	-	7,00	-	7,00	-	-
**2**	122,47	<0,001	4242,64	<0,001	0,004	362,04	<0,001	362,04	<0,001	0,037	5,00	<0,001	5,00	<0,001	0,625
**16**	69,28	<0,001	2323,79	<0,001	<0,001	362,04	<0,001	362,04	<0,001	0,02	6,00	<0,001	5,00	<0,001	0,041
**30**	7,75	<0,001	197,48	<0,001	<0,001	90,51	<0,001	362,04	<0,001	<0,001	6,00	0,002	5,00	<0,001	<0,001
**44**	0,53	<0,001	28,28	<0,001	<0,001	22,63	<0,001	181,02	<0,001	<0,001	6,00	0,008	6,00	<0,001	0,104
**58**	0,53	<0,001	11,83	<0,001	<0,001	11,31	<0,001	90,51	<0,001	<0,001	6,00	0,063	6,00	<0,001	0,054
**72**	0,13	<0,001	1,06	<0,001	<0,001	5,66	0,016	22,63	<0,001	<0,001	7,00	0,25	6,00	0,078	0,129
**86**	0,06	<0,001	0,28	<0,001	<0,001	5,66	0,188	22,63	<0,001	<0,001	6,00	0,375	6,00	0,016	0,098
**100**	0,06	0,009	0,13	<0,001	<0,001	5,66	1	11,31	<0,001	<0,001	7,00	0,25	6,00	0,008	0,055
**114**	0,06	0,055	0,13	<0,001	<0,001	5,66	1	5,66	0,01	0,005	7,00	0,109	6,00	0,125	0,844
**128**	0,06	0,055	0,13	<0,001	<0,001	5,66	1	5,66	0,02	0,01	7,00	0,313	6,00	0,063	0,375

On MPT, articaine abolished the difference to the control test result from the point of the 72 min (p > 0.05). The significant difference between control measurement and results of process of mepivacaine existed until the end of measurement (p < 0.05). Mepivacaine had a stronger influence (p > 0.05) over the entire time of measurement on MDT as well as on MPT; in direct comparison to articaine, relevant thresholds were increased significantly during the whole period of time. Almost over the entire measurement in VDT, mepivacaine had a stronger effect than articaine, whereas only two measurements showed a significant discrepancy between the two agents. Again, both pharmaceutical values of the control measurement were achieved for the measurement progress, whereas that proceeded faster for articaine (t = 58 min, p > 0.05) than for mepivacaine (t = 114 min, p > 0.05; Figure 3).

### Testing the pulp sensibility

**Figure 4:**
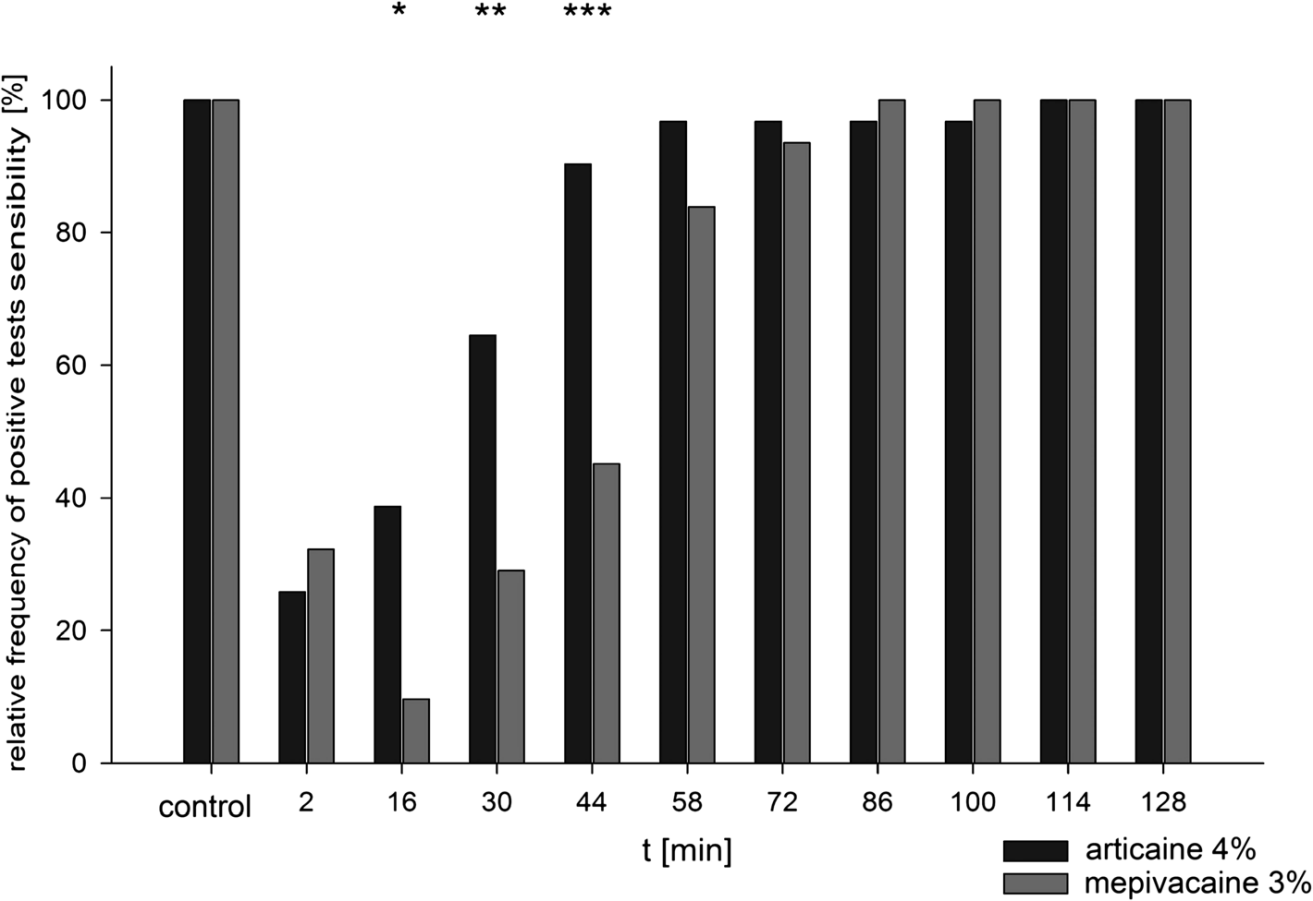
**Representation of the relative frequency of positive samples to the sensitivity of the individual measuring time points for both local anesthetics in direct comparison.**

The measurements of testing pulp sensitivity of the anesthetized tooth showed significant differences between the two local anesthetics at three points of measurement. The success rate for maxillary buccal infiltration in canine area to produce pulpal anesthesia using articaine was 74.19%, while the success rate was 90.32% using mepivacaine solution. By using mepivacaine it was possible to anesthetize more patients at the treated teeth region for a longer time. For both agents returning to baseline pulp sensibility was obtained within the testing period, while this point was reached earlier using articaine as agent (t = 44 min, p > 0.05) than using mepivacaine as agent (t = 58 min, p > 0.05; Figure 4).

The results of duration of pulp sensitivity testing showed a similar course. A period in which a positive sensitivity testing was considerably increased was 16 to 58 minutes for mepivacaine. At this period the two drugs showed differences, whereas the minutes 30 and 44 were significant (p < 0.05). In both preparations no significant differences between the measurement of control and the course measurements, starting at 86th minute, were observed (p > 0.05).

## Discussion

### Method and external parameter

In this prospective study, a non-randomized controlled clinical trial, 31 patients (16 male, 15 female patients) were tested with QST while undergoing a short-time dental treatment under local anesthesia in area of maxillary canine.

As previous studies proved that a difference in the perception of sensitive stimuli between healthy men and women does exist in this study, attention was paid to a homogeneous distribution of gender in order to avoid prejudicing the results. Furthermore, the age lowers the pain threshold (higher sensitivity) and leads to a slightly different reception of hot and cold stimuli [20,24,27,28]. In this study, the age of patients was limited in order to avoid any influences on the measurement results.

In previous studies QST was used successfully in the infraorbital region as detection method [20,24]. This study discovered the investigation area for the examination as a suitable method. This is attributable to the fact that on the one hand the region of canine represents an extremely sensitive area of innervation (N.trigeminus, N.infraorbitalis V2) and on the other hand the requirement of reproducibility is satisfied since the canine-region is used, despite individual anatomical differences, as a reference point for the repeated application of the measuring instruments. A difference in perception between the left and right sides of the examined QST parameters could not be determined [24,29].

In this study attention was particularly paid to the side distribution using local anesthetics. Both preparations were used on both sides for equal times. Thereby side distribution did not have an impact on the results.

In dentistry, choosing the right local anesthetics is of huge importance, the aspects of biocompatibility, tolerability and allergic potential should be considered [25,30]. The effect of local anesthetics on sensitivity can be evaluated in different ways. Commonly used methods examine the sensitivity of the anesthetized tooth, i.e. by checking the sensibility of the nerve fibers within the pulp. In order to check them, physical methods which involve thermal- (cold or heat application), electrical- and electro-optical measurement are used [26]. But those methods do not offer a review of the sensitivity of soft tissue, since it is also numbed by the used agent. Nevertheless the anesthesia of soft tissue, which most often affects the patient beyond the dental treatment, is an inevitable side effect of local anesthesia in dentistry and should be limited temporally, if possible. Especially for minor procedures, such as a routine-small-non surgical dental treatment, a discrepancy between the time of dental treatment implementation and the effect of anesthesia within the treated area as well as on the soft tissue does exist. Within currently available methods to study the function of sensory Aδ-, Aß- and C- fibers, QST is a reliable and repeatable method [20,24,31].

A comparison between different local anesthetics of equal amounts is restricted, since different anesthetic-agents have various relative efficacies and various intrinsic activities.

Local anesthetics with an approximately similar relative efficacy and intrinsic activity, but a different molecular weight, can only be compared theoretically in equimolar solutions. Studies working with equimolar solutions are only partially applied in practical clinical use. The applicability of data obtained in this study has priority for dental daily routine. In the present study, always the same volume (1.00 ml) of the different anesthetic-agent was applied, despite the fact that different doses were applied to each side. The used volume was taken to ensure both: first of all that local anesthesia of tooth for the short dental procedure was adequate and furthermore that the investigation of the anesthetic effect on soft tissue performing QST was in a realizing range (128 minutes) for the patient.

### Action of local anesthetics on pulpal sensitivity/effect of the local anesthetic to the QST parameters and their associated nerve fibers

The investigation of the effect of anesthesia on pulp sensibility showed that there are significant differences between the compared preparations. Mepivacaine is the stronger preparation in the applied volume. It has both, a stronger effect in terms of absolute number of negative samples than articaine, as well as the time until positive samples of sensitivity are measured. Articaine, however, shows a faster onset of action associated with a short duration of action.

Articaine has a higher number of negative samples of detectable sensitivity immediately after the application. This observation is also consistent with results of other studies. Because of a good bone- and soft tissue penetration of the active agent articaine, a possible reason for the rapid onset of action can be proven in different studies [32-34]. Mepivacaine reaches the maximum effect at a later point of time (t = 16 min).

*In vitro* studies confirm a consistent finding that articaine seems to be superior in the anesthetic efficacy. In an *in vitro* study Potocnik et al. were able to show that at sural nerve of a rat articaine 4% anesthetic solution was more effective than a lidocaine 4% or mepivacaine 3% solution [35]. This result has also been demonstrated in other *in vitro* studies on isolated nerves of frogs and rats [36].

In a comparative clinical trial Cowan could show that in a dental infiltration anesthesia with equal volumes (1.00 ml) of anesthetic agents the anesthetic effect of articaine without an added vasoconstrictor is less than the anesthetic effect of lidocaine 2% and mepivacaine 3% [15]. A similar result was also obtained by Winther and Nathalang in a comparative study. They discovered that the epinephrine-free solution could cause lack of adequate clinical analgesia in both, 2% and in 4% concentration, in contrast to epinephrine-containing articaine solutions [37]. In a comparison of 1% solutions of both local anesthetics Sommer et al. showed that mepivacaine had almost the doubled action time as articaine [38]. A possible reason may be the low but existing vasoconstrictor effect of mepivacaine and the pronounced vasodilatory effect of articaine. These substance properties play only a minor role in the *in vitro* model of the isolated nerve, whereas they are quite detectable in clinical use because of the vascularized tissue [1]. In contrast to that, Rahn et al. demonstrated that a 2% articaine-epinephrine-free solution compared to standard articaine (4% articaine with epinephrine solution of 1/200.000) can be perfectly used in clinical routine and has even be proven in surgical intervention. This performance was also confirmed by Kämmerer et al. who successfully used a 4% articaine-epinephrine-free solution for tooth extraction in the mandible [14,39]. A comparison of dental anesthetic success of local anesthetics between this study and other studies shows that the observed anesthesia success in this study agrees with the values obtained in other studies. The anesthesia success for mepivacaine is assessed as high (t = 16 min, anesthesia success 90.32%) and for articaine as moderate (t = 2 min, anesthesia success 74.19%). Moore et al. were able to achieve a comparative success with articaine 4% anesthesia without epinephrine in a maxillary infiltration anesthesia (1.00 ml) of 75.8%. This almost agrees with the results of this study [40-43].

The study demonstrates that the applied amount of 1.00 ml of the anesthetic agent articaine is sufficiently high to achieve adequate success of anesthesia during small dental procedures.

The results of the QST parameters permit conclusions of certain nerve fibers [18,31]. Differences of the two preparations are obvious.

The two preparations show significant differences of the individual measuring times to each other and also of the control value compared to the values of each continuing measurements.

No significant difference between the control values and the measurement values of articaine in five of seven tests could be observed. This was only achieved at two of seven test parameters by mepivacaine. However, a differentiated end of the blockade and the regeneration of individual nerve fibers from the active ingredient of the local anesthetic are not evident. Accordingly, the sensitivity to the local anesthetic does not only depend on the diameter of the individual nerve fibers, but notably on the choice of the active substance and its physicochemical properties [44]. In opposite to the conventional amide anesthetics mepivacaine, which is only degraded in the liver, articaine, being metabolized in the liver and in plasma by Pseudocholinesterasen, shows a short interference of the thresholds which were measured. Furthermore, plain articaine has its strongest effect just after the injection at the first measurement point (based on the sensitivity of the tooth anesthetized). Whereas this can firstly be registered a measurement point later in the case of mepivacaine. Thus, the excellent tissue penetration and rapid onset of action can be confirmed for the active agent articaine, which is awarded in various studies. Both factors are based on the physicochemical properties, in particular increased by the thiophene lipophilicity. This allows a more efficient diffusion of articaine through soft tissue than other local anesthetics [32-34].

### Aβ- fibers (MDT, VDT)

The study of myelinated Aβ-fibers took place via QST parameters MDT and VDT. This demonstrates that there are significant differences at all measuring times between the two preparations at MDT. The active agent mepivacaine showed a stronger influence on MDT as articaine at all measuring times. A significant difference between the control value and the value of the course of the active agent mepivacaine was detectable on the entire range of measurement. In contrast to that, articaine did not show any significant difference at the end of the measurement time (114 minutes). In various studies, which were already carried out on the face, MDT presented to be particularly sensitive test parameters [18].

The local anesthetic effect on these test parameters might be the reason for the long time influence.

The VDT test parameters show significant differences of the two preparations concerning the measuring times of 16 and 30 minutes. Again, mepivacaine was more effective than articaine.

The comparison between the control values and the progress values showed no significant difference at the end of measurement for both active substances, although the end of significant differences was reached earlier by articaine than by mepivacaine. The results show that the myelinated Aβ-fibers, which are associated with the test parameters, recover very fast from the local anesthetic action. This agrees with previous studies [45,46]. Especially articaine seems to affect Aβ-fibers less than mepivacaine. However, it should be noted that there can be a transmission of the vibration to the maxillary bone protrusion [24]. This may lead to stimulation of unanesthetized areas, a distortion of the measured values and that VDT loses informative value compared with MDT.

### Aδ- fibers (CDT, MPT, HPT)

The test parameters associated with the Aδ-fiber activity show at almost all points of time significant differences between the two preparations. Thereby mepivacaine has a stronger effect on the test parameters than articaine.

The test parameters HPT and MPT do not show significant differences between the progress values and the control value for articaine within the study period of 128 minutes at the end of the measurements.

The results suggest that articaine has less influence on Aδ-fiber activity than mepivacaine. Also the regeneration of nerve fibers from the local anesthetic effect of articaine is faster than the regeneration from the effect of mepivacaine.

### C-fibers (WDT, CPT, HPT)

The test parameters associated with the C-fiber activity showed at almost all points of measurement a significantly stronger effect of mepivacaine compared to articaine. During the test a significant difference of both agents remains at WDT parameters until the end of the measurement period. However, a stronger influence of the test parameter was recorded for mepivacaine. Surprisingly, it appears that at CPT the significant difference between the value of measurements and the control value was canceled for both agents. This occured faster in the case of articaine than in the case of mepivacaine.

As far as HPT is considered, only articaine achieved the end of significant difference during the measurement, whereas mepivacaine still showed significant discrepancy during the measurement range to the end of measurement.

## Conclusion

The aim of the study was to investigate the analgesic effect of tooth as well as the anesthetic numbing effect of soft tissue which is caused by local anesthetics as 4% articaine and 3% mepivacaine which do not contain a vasoconstrictor. The results of this study show that mepivacaine causes a longer analgesia to the anesthetized tooth and has a stronger influence on the investigated thermal and mechanical test parameters at all measurement points of time. Plain articaine 4% shows an earlier onset of action associated with a shorter time of activity in comparison to plain mepivacaine 3%. This can result in more frequent injections of the local anesthetic agent, if articaine is used. In addition to this articaine shows a significant low-graded effect on the tested nerve-fibers and therefore a least affected anesthesia to the patient. Plain articaine 4% is very close to the demands of a differentiated anesthesia. The clinical use of an epinephrine-free anesthetic solution can be stated as possible option in short dental routine treatments to the frequently used vasoconstrictor containing local anesthetics.
